# Evolution of Flavylium-Based Color Systems in Plants: What Physical Chemistry Can Tell Us

**DOI:** 10.3390/ijms22083833

**Published:** 2021-04-07

**Authors:** Fernando Pina, Alfonso Alejo-Armijo, Adelaide Clemente, Johan Mendoza, André Seco, Nuno Basílio, António Jorge Parola

**Affiliations:** 1 LAQV—REQUIMTE, Departamento de Química, Faculdade de Ciências e Tecnologia, Universidade Nova de Lisboa, 2829-516 Caparica, Portugal; aaa00010@red.ujaen.es (A.A.-A.); johanmen2a@gmail.com (J.M.); am.seco@campus.fct.unl.pt (A.S.); nuno.basilio@fct.unl.pt (N.B.); ajp@fct.unl.pt (A.J.P.); 2 cE3c—Centre for Ecology, Evolution and Environmental Changes, Faculdade de Ciências, Universidade de Lisboa, Campo Grande, 1749-016 Lisboa, Portugal; maclemente@fc.ul.pt

**Keywords:** anthocyanins, 3-deoxyanthocyanins, auronidins, color of plants evolution

## Abstract

Anthocyanins are the basis of the color of angiosperms, 3-deoxyanthocyanins and sphagnorubin play the same role in mosses and ferns, and auronidins are responsible for the color in liverworts. In this study, the color system of cyanidin-3-*O*-glucoside (kuromanin) as a representative compound of simpler anthocyanins was fully characterized by stopped flow. This type of anthocyanin cannot confer significant color to plants without intra- or intermolecular interactions, complexation with metals or supramolecular structures as in *Commelina communis.* The anthocyanin’s color system was compared with those of 3-deoxyanthocyanins and riccionidin A, the aglycone of auronidins. The three systems follow the same sequence of chemical reactions, but the respective thermodynamics and kinetics are dramatically different.

## 1. Introduction

**Scheme 1:**
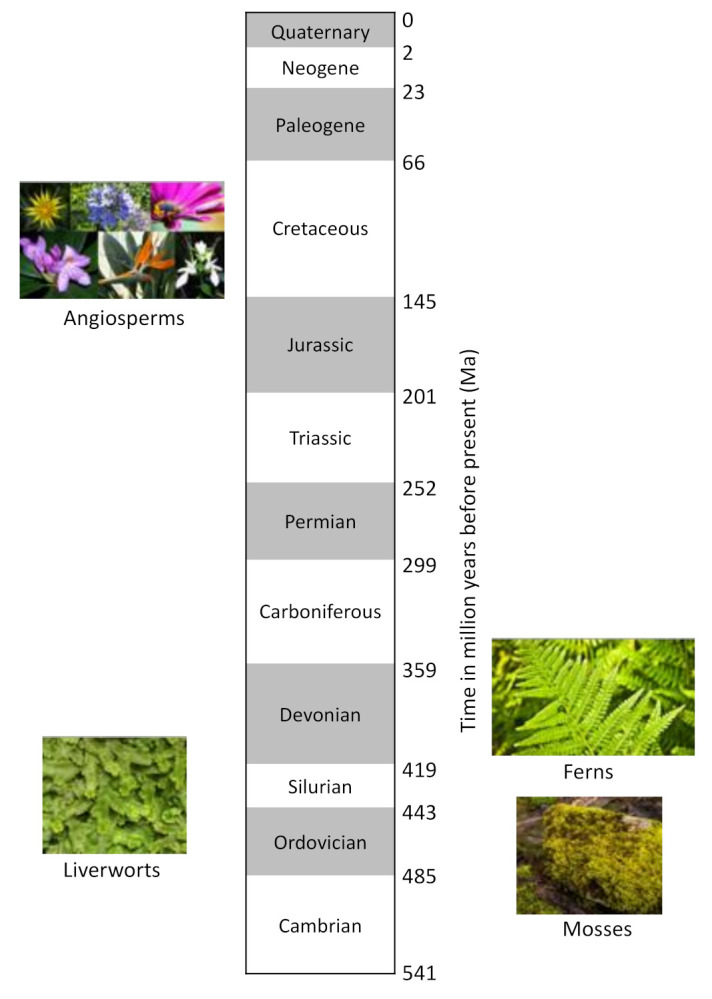
Estimated origin of liverworts, mosses, ferns, and angiosperms. Jason Hollinger, Charles Peterson, Peter and Joyce Grace, Sarah Vanderweele, Camelia TWU, and Rafael Medina are credited for the photographs presented in Scheme 1, depicting several plant species.

The thesis presented in this work is that the anthocyanin system that confers color to angiosperms, starred by different molecules, was previously used by liverworts, mosses, and ferns. By the anthocyanin system, we mean the flavylium cation, quinoidal base, hemiketal, and *cis-* and *trans*-chalcones, and the chemical reactions that interconvert them. The estimates for the origin of mosses fall within the Cambrian to Ordovician period [1], and those of liverworts within the Ordovician to Early Devonian period [2,3], but several competing hypotheses have hindered estimates of a consensual age for these groups [4]. Ferns originated in the Early-Mid Devonian [5] and angiosperms during the Lower Cretaceous, possibly earlier (Scheme 1) [6]. The color systems used by these plants are based on furanoflavylium cations (liverworts), 3-deoxyanthocyanidins and sphagnorubin (mosses and ferns), and anthocyanins (angiosperms). The color system of anthocyanins is unique in versatility when compared with those of furanoflavylium and 3-deoxyanthocyanins.

**Scheme 2:**
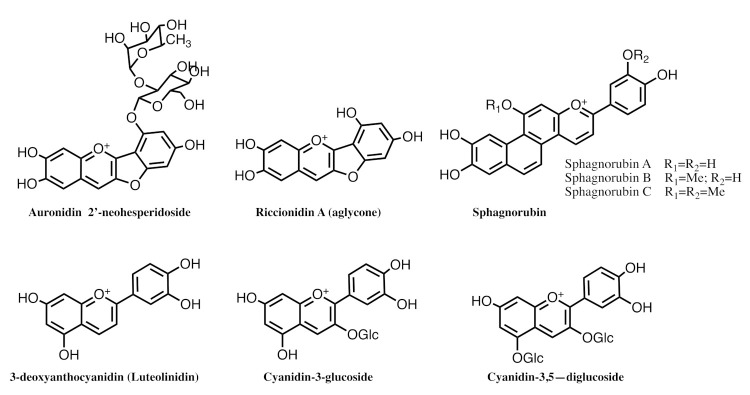
The flavylium cation of auronidins, 3-deoxyanthocyanins, sphagnorubins, and anthocyanins.

Color is ubiquitous in nature and anthocyanins are the largest class of water-soluble compounds present in the plant kingdom responsible for a range of colors in plants, from orange-red to purple-blue hues. Anthocyanins are glycosylated forms of anthocyanidins, normally occurring as 3-glycosides or 3,5-diglycosides (Scheme 2). Different types of monosaccharides (e.g., glucose, galactose, rhamnose, and arabinose) and disaccharides (e.g., rutinose, sambubiose, and sophorose) can be attached to the flavylium core, and these sugars can also be acylated with cinnamic acids (e.g., caffeic, coumaric, ferulic, and sinapic acids) or aliphatic acids (e.g., acetic, malic, oxalic, and succinic acids) [7]. Related to anthocyanins are 3-deoxyanthocyanidins, found in mosses and ferns, sphagnorubins found in peat moss [8,9], and auronidins (furanoflavylium derivatives) (Scheme 2) [10]. Andersen and Davies, questioned whether “auronidins might contribute to the remarkable ability of liverworts to survive in extreme environments on land, and their discovery calls into question the possible pigment status of the first land plants” [10].

We devoted the past two decades to the study of the kinetics and thermodynamics of the natural anthocyanins, 3-deoxyanthocyanins, and the related synthetic flavylium compounds. Following these studies, we investigated the kinetics and thermodynamics of furanoflavylium compounds [11,12] and, more recently, riccionidin A, the aglycone of the auronidin-2′-neohesperidoside reported by Andersen and Davies [10,13]. In this work we investigate the three color systems used by plants auronidins, 3-deoxyanthocyanins, and anthocyanins—providing physicochemical arguments to suggest that the three color systems in these families of plants had a chemical evolution. Some similarities and differences between the three systems have previously been reported by some of the authors [13]. In this work, we focus essentially on the higher potentialities of the anthocyanin color system when compared to the other two systems. The newly reported theoretical corpus based on the reverse pH jumps followed by stopped flow, which has been used to characterize malvidin-3-*O*-glocoside (oenin) [14], was applied to cyanidin-3-*O*-glucoside (kuromanin). This work reflects the chemical aspects of the plants color systems and do not regards phylogenetic issues, which can be found in specialized publications [15,16].

## 2. Results and Discussion

### 2.1. The Color System of Anthocyanins. The Case of Cyanidin-3-O-Glucoside (Kuromanin)

**Scheme 3:**
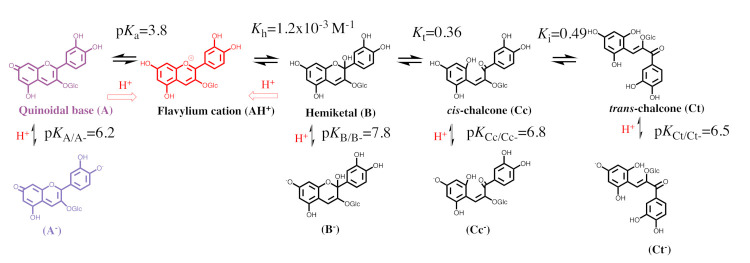
Multistate of chemical species of the anthocyanin kuromanin in acidic medium. At sufficiently high proton concentrations, the system converges to the flavylium cation: *K*_n_ = *k*_n_/*k*_−n_ (n = a, h, t, i).

The color system of kuromanin is shown in Scheme 3.

The flavylium cation is the sole species at pH ≤ 1. When the pH is increased to moderately acidic solutions (hereon referred to as direct pH jumps), other species are formed in the following sequence: quinoidal base, hemiketal, *cis*-chalcone, and *trans*-chalcone. At higher pH values the anionic, or multi-anionic, analogues of the neutral species are formed, depending on the pH.

**Figure 1:**
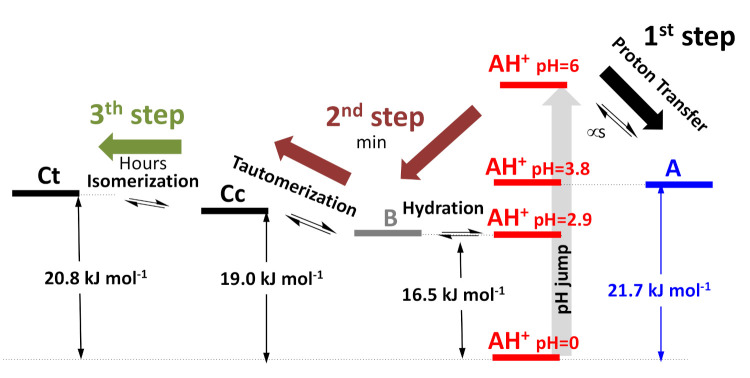
Energy level diagram for kuromanin in acidic medium. The sequence of reactions after a direct pH jump is shown.

The kinetic processes towards the equilibrium after a direct pH jump are illustrative, see Figure 1. Proton transfer is clearly the faster step of the kinetics and occurs during the mixing time of the stopped flow (a few milliseconds). During the following steps, **AH^+^** and **A** behave as a single species with the respective ratio equal to [**A**]/[**AH**^+^] = *K*_a_/[H^+^]. The next step is the formation of the hemiketal, **B**, through the hydration of **AH^+^** (min), followed by the ring opening to yield *cis*-chalcone, **Cc**, (ms/s). The fact that the quinoidal base does not hydrate in acidic medium is a breakthrough discovery [17] crucial for the comprehension of the behavior of anthocyanins and related compounds. Consequently, the evolution of the system towards the pseudo-equilibrium (in acidic to moderately acidic medium) occurs from the flavylium cation hydration. The isomerization to yield *trans*-chalcone, **Ct**, in anthocyanins takes place in hours. When the system is equilibrated in moderately acidic pH values, addition of acid to reach pH ≤ 1 (reverse pH jumps) restores the flavylium cation. This is the basis for applying the reverse pH jumps followed by stopped flow.

The thermodynamic behavior of anthocyanins can be represented by an energy level diagram [18,19] (Figure 1) or by the mole fraction distribution as a function of pH (see below) [20,21]. Both representations can be extended to the anionic forms obtained through deprotonation of the hydroxyl substituents in neutral and basic solutions. However, the energy level diagram becomes more complex and difficult to interpret when the anionic species are included and, as a result, is usually used to describe the equilibrium of the first row of Scheme 3.

### 2.2. Anthocyanin Multistate

The equilibrium involving anthocyanins and presented in Scheme 2 and Figure 1 is accounted for by Equations (1)–(8):

**AH^+^** + H_2_O ⇌ **A** + H_3_O^+^    *K*_a_  proton transfer (1)

**AH^+^** + 2H_2_O ⇌ **B** + H_3_O^+^   *K*_h_  hydration (2)

**B** ⇌ **Cc**    *K*_t_  tautomerization (3)

**Cc** ⇌ **Ct**    *K*_i_  isomerization (4)

Extending to higher pH values:

**A** + H_2_O ⇌ **A*^−^*** + H_3_O^+^    *K*_A/A***−***_  proton transfer (5)

**B** + H_2_O ⇌ **B*^−^*** + H_3_O^+^    *K*_B/B***−***_  proton transfer (6)

**Cc** + H_2_O ⇌ **Cc*^−^*** + H_3_O^+^    *K*_Cc/Cc***−***_  proton transfer (7)

**Ct** + H_2_O ⇌ **Ct*^−^*** + H_3_O^+^    *K*_Ct/Ct***−***_  proton transfer (8)

This complex system can be dramatically simplified to a polyprotic acid given by Equations (9)–(12):

(9) $${\mathrm{AH}}^{+}\;+\;H_{2}O\;⇌\;\mathrm{CB}\;+\;H_{3}O^{+}\;K_{a}^{\prime }=K_{a}+K_{h}+K_{h}K_{t}+K_{h}K_{t}K_{i}$$

**CB** = [**A**] + [**B**] + [**Cc**] + [**Ct**] (10)

(11) $$\mathrm{CB}\;+\;H_{2}O\;⇌\;{\mathrm{CB}}^{-}\;+\;H_{3}O^{+}\;K_{a}^{\prime \prime }=\frac{K_{A/A^{-}}K_{a}+K_{B/B^{-}}K_{h}+K_{\mathrm{Cc}/{\mathrm{Cc}}^{-}}K_{h}K_{t}+K_{\mathrm{Ct}/{\mathrm{Ct}}^{-}}K_{h}K_{t}K_{i}}{K_{a}^{\prime }}$$

**CB**^−^ = [**A**^−^] + [**B**^−^] + [**Cc**^−^] + [**Ct**^−^] (12)

The fact that this complex system can be simplified considering the flavylium cation in equilibrium with its conjugated forms **CB** and **CB^−^** allows the mathematical treatment to be simplified.

In anthocyanins and many other flavylium-derived multistate of species, the isomerization is clearly the slowest process of the kinetics and a pseudo-equilibrium can be defined as a transient state obtained before significant formation of *trans*-chalcone (Equations (1)–(3) and Equations (5)–(7)) and the respective pseudo-equilibrium constants defined by Equations (13)–(16):

(13) AH+ + H2O ⇌CB^+ H3O+  Ka^=Ka+Kh+KhKt

**CB^^^** = [**A**] + [**B**] + [**Cc**] (14)

(15) CB^ + H2O ⇌ CB^− + H3O+Ka^^=KA/A−Ka+KB/B−Kh+KCc/Cc−KhKtKa^

**CB**^^–^ = [**A**^−^] + [**B**^−^] + [**Cc**^−^] (16)

During the 3rd step of the kinetics in Figure 1, all species except **Ct** are in equilibrium.

### 2.3. How to Calculate All Equilibrium Constants. Reverse pH Jumps: A New Paradigm

**Figure 2:**
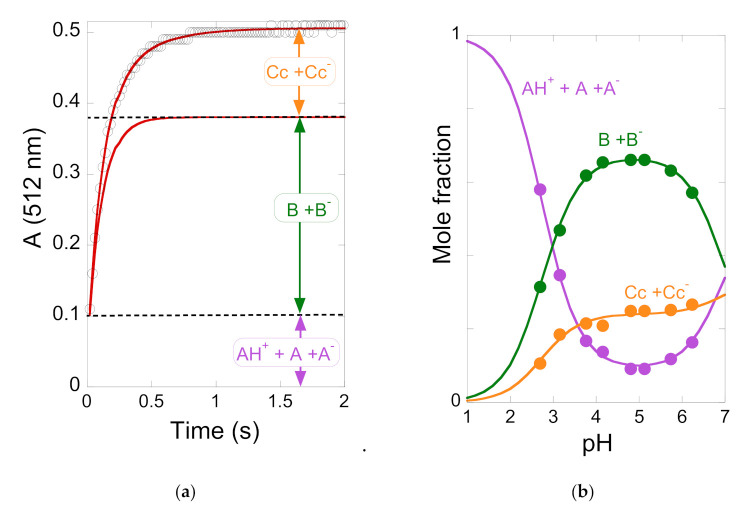
(**a**) Trace of a reverse pH jump of a pseudo-equilibrated solution of kuromanin at pH = 5.1 followed at 512 nm by stopped flow showing the three observed amplitudes; (**b**) mole fraction distribution at the pseudo-equilibrium, obtained after reverse pH jumps as in (**a**) for different pH values.

**Figure 3:**
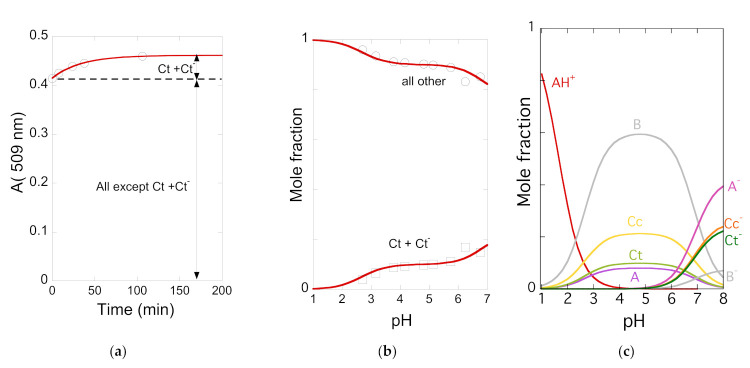
(**a**) Trace of a reverse pH jump of an equilibrated solution of kuromanin at pH = 5.1 followed at 509 nm by standard spectrophotometer; (**b**) mole fraction distribution of *trans*-chalcone at the equilibrium; and (**c**) mole fraction distribution of all AH^+^, CB, and CB^−^ species at the equilibrium.

The most accurate method to determine the equilibrium constants reported in Scheme 3 is based on the reverse pH jumps (defined by the addition of acid to ensure pH ≤ 1 of equilibrated solutions, or pseudo-equilibrated, at higher pH values) [22] monitored by stopped flow [23]. In Figure 2a, the trace of a typical reverse pH jump is shown. Three amplitudes are observed. The first is due to the flavylium cation absorption resulting from the conversion of all quinoidal bases (neutral and anionic) during the mixing time of the stopped flow together with some flavylium that is in equilibrium prior to the pH jump (for lower pH values). The amplitude of the faster kinetic step results from the conversion of hemiketal (neutral and anionic) into flavylium cation, because at pH ≤ 1 the change of regime causes the hydration/dehydration to be faster than the tautomerization [22,24]. The final amplitude corresponds to the formation of more flavylium cation from *cis*-chalcone (neutral and anionic) via hemiketal. Normalization of these amplitudes to the unit directly yields the mole fraction distribution of these species at the pseudo-equilibrium as shown in Figure 2b. The experimental data obtained for kuromanin in Figure 2 and Figure 3 was used to calculate the equilibrium constants reported in Figure 1 according to the mathematical expressions derived in [23]. Once these constants were obtained, the energy level diagram can be constructed considering that Δ*G*° = *RT* ln*K*, where Δ*G*° is the Gibbs free energy, *R* is the ideal gas constant, *T* is the absolute temperature (Kelvin degrees), and *K* is the equilibrium constant of the chemical reaction [18,19].

The mole fraction of **Ct** and **Ct^−^** was calculated by following the reverse pH jumps through a standard spectrophotometer, as shown in Figure 3a. Extending to a series of pH jumps from different pH values, the mole fraction distribution of **Ct** was calculated (Figure 3b), and the mole fractions of **AH^+^**, **A+A^−^**, **B+B^−^**, and **Cc+Cc^−^** were recalculated for the equilibrium, discounting the equilibrium of **Ct** and **Ct^−^** (Figure 3c). Table 1 shows the equilibrium constants of kuromanin and the mole fraction distribution of all species at the equilibrium are presented in (Figure 3c). It should be emphasized that the constants regarding the anionic species have higher uncertainty because of the propagation of the error law (depending on the equilibrium constants of the neutral species) and due to the instability of the simpler anthocyanins at higher pH values, in particular, to determine the mole fractions of **Ct**.

### 2.4. Limits of Anthocyanins to Confer Colour

**Figure 4:**
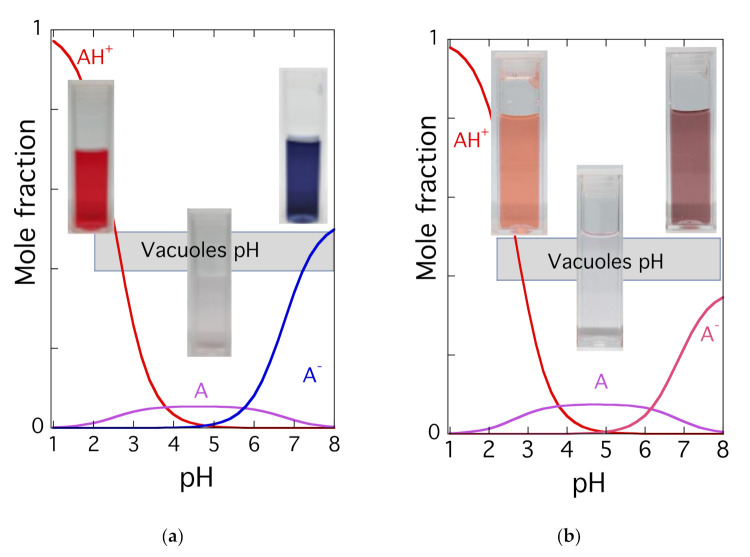
(**a**) Mole fractions at the pseudo-equilibrium of the colored forms of oenin (**b**) and kuromanin; in this case the blue color is observed only for the dianionic quinoidal base (not shown).

**Figure 5:**
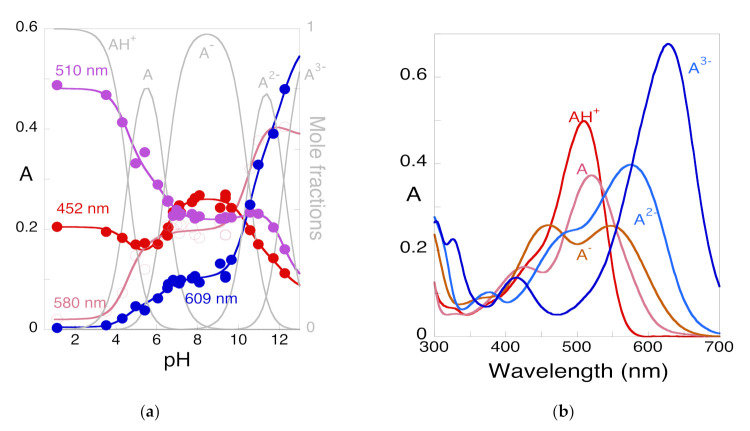
(**a**) Mole fraction distribution of the kuromanin (1.5 × 10^−5^ M) flavylium cation and quinoidal base species obtained 10 ms after a direct pH jump followed by stopped flow; p*K*_a1_ = 4.0; p*K*_a2_ = 6.5; p*K*_a3_ = 10.0; p*K*_a4_ = 12.2; (**b**) Spectral decomposition achieved for obtained p*K*_a_ values.

Anthocyanins are located in the vacuoles. The pH of the vacuoles may vary considerably, but a pH of approximately 5 is often observed [25]. However, the pH of the vacuoles could be as low as 2.0 in citrus fruits [26] and 7.7 or more in some flowers, Figure 4 [27]. In anthocyanins, the colored species are flavylium cation and quinoidal bases (neutral and anionic). In the case of simple anthocyanins such as malvidin-3-*O*-glucoside (oenin) and cyanidin-3-*O*-glucoside (kuromanin), the appearance of color at the equilibrium is only observed for the flavylium cation at very low pH values, and at moderately acidic pH values the mole fraction of the quinoidal base is low. Consequently, neither flavylium cation nor quinoidal base can confer significant color at moderately acidic solutions, as shown in the photos of Figure 4. Curiously, at pH values close to neutrality, the color of the anionic quinoidal base is accessible but at these pH values these anthocyanins are unstable. It is also interesting to note that, despite the generally accepted view by the scientific community and contrary to what can be observed for oenin, the ionized quinoidal base (**A**^−^) of kuromanin seems to not confer the blue color to the solution (see Figure 4). To investigate the phenomenon in more detail, a series of direct pH jumps from pH = 1 to higher pH values (up to pH = 12) were performed using stopped flow to calculate the four deprotonation constants. This technique allows for the fast (ca. 10 ms after the pH jump) acquisition of the absorption spectra, which are necessary to investigate the acid-base equilibria without interference of the hydration and subsequent reactions that lead to color fading. Figure 5a shows the plots of absorbance versus pH, at four different wavelengths, that were used to obtain the respective p*K*_a_ values for the formation of the quinoidal base and its ionized forms from the flavylium cation. Using this methodology, the following values were obtained: p*K*_a1_ = 4.0; p*K*_a2_ = 6.5; p*K*_a3_ = 10.0; and p*K*_a4_ = 12.2. These values compare to those obtained for oenin: p*K*_a1_ = 3.9; p*K*_a2_ = 6.3; and p*K*_a3_ = 8.4 [28]. Although the first two values are reasonably similar, the p*K*_a3_ for the formation of the di-ionized quinoidal base is considerably higher for kuromanin. By combining the pH-dependent mole fraction distribution of the flavylium cation and all quinoidal bases with the respective spectral data, it is possible to obtain the spectra of the five species by spectral decomposition (Figure 5b). The results clearly show that the spectrum of A^−^ cannot account for the blue color (in contrast with **A**^−^ of oenin, which is relatively red-shifted) and that this color can only be expressed by A^2−^ and A^3−^, which appear only at very basic pH values. As these conditions do not seem feasible in biological systems, the results suggest that kuromanin can only express the blue color when complexed with metal cations or by another mechanism that may shift the third and fourth p*K*_a_ to lower values.

To overcome the limits of simpler anthocyanins to confer color, different strategies are used in nature. One of the most studied is copigmentation, defined by Robinson as the modifications of the anthocyanin absorption spectrum caused by colorless compounds, such as amino acids, sugars, and flavonoids [1,2,29,30]. Copigmentation could be intermolecular and/or intramolecular, as observed in acylated anthocyanins [31,32,33]. Other strategies used in nature are self-association [34] and metal complexation [35]. Summarizing, the stabilization of the colored species is usually driven by several noncovalent interactions, such as hydrogen bonding, van-der-Waals, π–π stacking, metal–ligand interactions, and hydrophobic-driven associations [33].

### 2.5. The Kinetics of the Multistate

The magnitude of the rates of the interconversion between the several species of the anthocyanins multistate is shown in Figure 1. After a direct pH jump, for example, to pH = 6, the quinoidal base is formed, with a rate that is faster than the mixing time of the stopped-flow, requiring other techniques, such as temperature jumps [17] or flash photolysis [36], to be observed. In the following kinetic steps **AH^+^** and **A** are in equilibrium, at the respective ratio equal to [**A**]/[**AH**^+^] = *K_a_*/[H^+^]. The disappearance of **AH^+^** and **A** towards the equilibrium is a biexponential process in which the 2nd and 3rd steps exhibit different lifetimes, respectively minutes and hours. As the tautomerization is faster than the hydration at the pH values reached in direct pH jumps, the second step is controlled by the last.

The expression that accounts for the 2nd step was achieved in a straightforward manner considering that the species **AH^+^** and **A** on one side and **B** and **Cc** on the other are in fast equilibrium during the hydration step, in Equation (5) [37]:

(17) $$k_{2nd(direct)}=\chi_{AH+}k_{h}+\chi_{B}k_{-h}\left[H^{+}\right]=\frac{\left[H^{+}\right]}{\left[H^{+}\right]+K_{a}}k_{h}+\frac{1}{1+K_{t}}k_{-h}\left[H^{+}\right]$$

Here, $\chi_{AH+}$ and $\chi_{B}$ are the mole fraction of **AH^+^** in its equilibrium with **A** and the mole fraction of **B** in its equilibrium with **Cc,** respectively. At the end of the 2nd step, the system reaches the pseudo-equilibrium. The 3rd step is controlled by the isomerization and, due to the difference between the rates between of the 2nd and 3rd steps, **AH^+^**, **A, B,** and **Cc** can be considered to be in equilibrium (that was defined as pseudo-equilibrium):

(18) $$k_{3rd(direct)}=\chi_{Cc}k_{i}+k_{-i}=\frac{K_{h}K_{t}}{\left[H^{+}\right]+K_{a}+K_{h}+K_{h}K_{t}}k_{i}+k_{-i}$$

where *χ_Cc_* is the mole fraction of Cc at the pseudo-equilibrium.

Some conclusions may be drawn regarding the thermodynamics and kinetics of the anthocyanin system. At the equilibrium, the red, purple, and blue colors are potentially available, but some strategies should be used by the plants to give expression to these colors; for example, intramolecular and/or intermolecular copigmentation in acylated anthocyanins and supramolecular structures such as the one that gives the blue color to *Commelina communis*, as reported by Nagoya’s group [35,38].

### 2.6. The Colour System of 3-deoxyanthocyanins

**Scheme 4:**
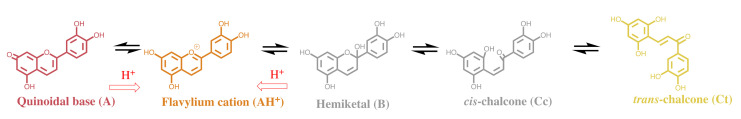
Kinetic scheme of luteolinidin.

**Scheme 5:**
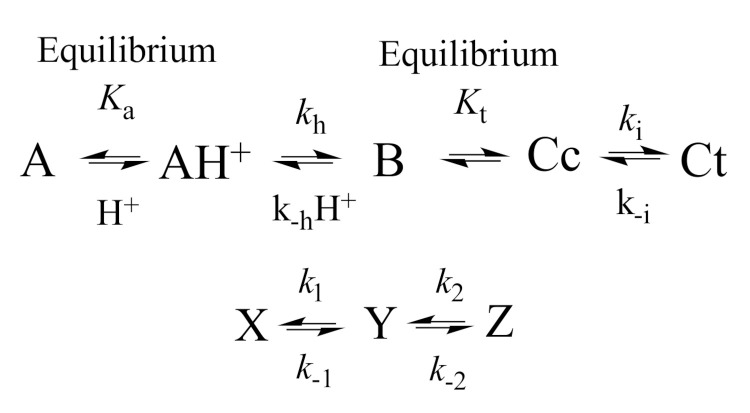
Kinetic scheme of a flavylium multistate lacking the pseudo-equilibrium.

As shown in Scheme 4, 3-deoxyanthocyanins follow the same multistate of species of anthocyanins but the isomerization is not the slowest kinetic step towards equilibrium. The situation is summarized in Scheme 5 [37].

In a direct pH jump, **AH^+^/A** conversion into **Ct** occurs via a single step of pseudo-first order kinetics. Considering **AH^+^** in fast equilibrium with **A** (X) on one side and **B** in fast equilibrium with **Cc** (**Y**) on the other to produce **Ct** (**Z**), a mechanism equivalent to a reversible kinetic scheme involving three species is required. When the steady state approach is applied to **Y**, Equation (19) can be derived [37]:

(19) $$k_{bell}=\frac{\frac{\left[H^{+}\right]}{\left[H^{+}\right]+K_{a}}K_{h}K_{t}k_{i}+k_{-i}\left[H^{+}\right]}{\left[H^{+}\right]+\frac{k_{i}K_{t}}{k_{-h}}}$$

**Figure 6:**
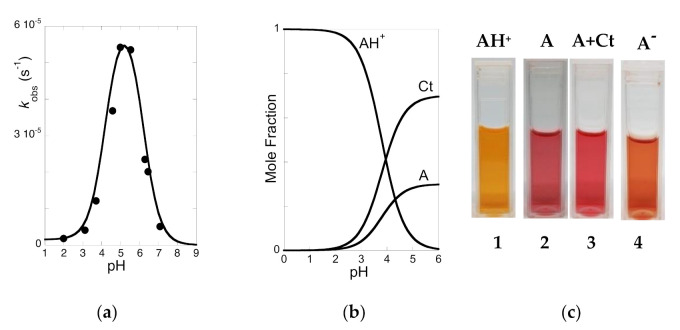
(**a**) Bell-shaped curve of the reaction towards equilibrium versus the pH of luteolinidin at 2.5 × 10^−5^ M in ethanol:water (1:1). Fitting was performed using Equation (10) for *K*_h_*K*_t_*K*_i_ = 4 × 10^−9^ M^−1^s^−1^; *K*_t_/*k*_i_/*k*_-h_ = 6.5 × 10^−7^ M^−1^s^−1^; *k*_-i_ = 1.5 × 10^−6^ s^−1^; and p*K*_a_ = 3.8. (**b**) Mole fraction distribution of the species at equilibrium, which basically consists in a quinoidal base in equilibrium with *trans*-chalcone in **CB**; and (**c**) (**1**) flavylium cation at pH = 1.0. (**2**) Quinoidal base immediately after a pH jump to pH = 5; (**3**) the same solution at the equilibrium; and (**4**) ionized quinoidal base immediately after a pH jump to pH = 8.0. Immediately after a direct pH jump to pH = 12, the color is dark orange (not shown). Adapted from [13].

Equation (19) yields a bell-shaped curve when represented as a function of pH. In Figure 6a, the bell-shaped curve of luteolinidin is shown.

For luteolinidin and related compounds, the rate constants of the reverse pH jumps are equal to the direct ones for the same final pH. Moreover, **B** and **Cc** are elusive species appearing at immeasurable concentrations in the stationary state. However, the mole fractions of **A** and **Ct** can be calculated from the data of the bell-shaped curve to obtain the respective mole fraction distribution from the ratios *K*_a_/*K*′_a_ and *K*_h_*K*_t_*K*_i_/*K*′_a_ (Figure 6b).

The comparison between the response to the kinetics of the color appearance and disappearance is discussed in the next section. No blue color can be achieved by 3-deoxyanthocyanins.

### 2.7. Comparison of the Colour System in Anthocyanins and 3-Deoxyanthocyanidins

The chemical structure of the cyanidin, the aglycone of kuromanin (anthocyanidin), when compared with luteolinidin, shows that they differ only in the hydroxyl in position 3. It is worth noting that cyanidin, like the other anthocyanidins, is very unstable in moderately acidic solutions [39], whereas luteolinidin [40] and sphagnorubin A [9], both found in mosses, are relatively stable. This suggests that it was not because of the stability that the hydroxyl was introduced into position 3 of anthocyanins. The reason was that this substitution gives rise to a red flavylium cation, a purple quinoidal base, and a bluish or blue (depending on the anthocyanin) anionic quinoidal base. The instability of anthocyanidins was solved by glycosylation of position 3 to give 3-*O*-anthocyanidins (anthocyanins). Glycosylation of position 5 that yields the diglucosilated anthocyanidins is not as significant for the stability.

**Scheme 6:**
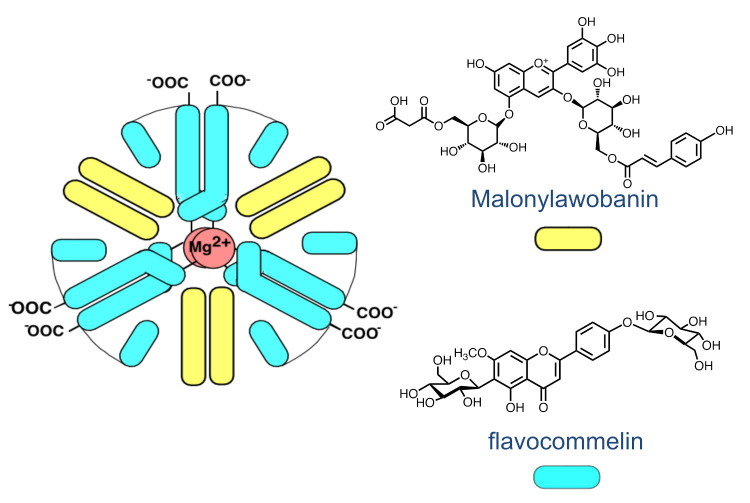
Sketch of the metalloanthocyanin responsible for the color of *Commelina communis*. The sketch was provided by Prof. Kumi Yoshida. The building blocks self-associate to produce the supramolecule in a bottom-up approach.

The quinoidal base of luteolinidin is red and the anionic quinoidal base is orange. There is no blue color. The blue color appears to be the most demanding in terms of the evolution. The excellent review by Yoshida et al. provides an overview of the structures used to express this color [35]. Intermolecular and intramolecular copigmentation (in acylated anthocyanins) and metal complexation are the strategies used to obtain a blue color. The most sophisticated structures (from the chemical perspective) are metaloanthocyanins, for example, the one that confers color to *Commelina communis* (Scheme 6). In this supramolecule, two metals, six anthocyanins, and six flavanones are organized in two parallel plans.

**Scheme 7:**
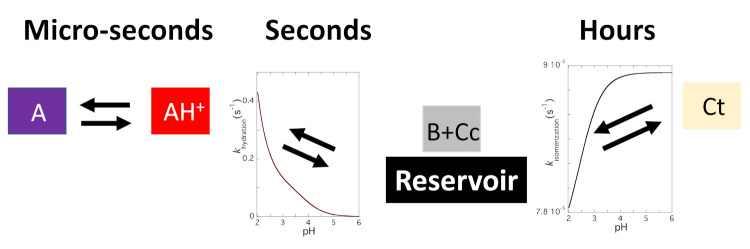
Conversion rates between the colorless reservoir in anthocyanins and the colored species.

**Scheme 8:**
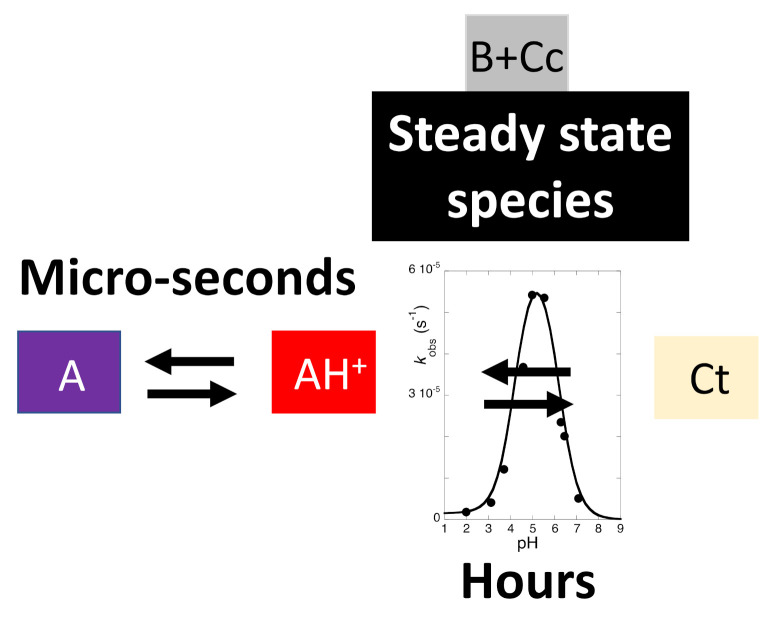
In 3-deoxyanthocyanins there is no reservoir of colorless species. **B** and **Cc** are the steady state species with undetectable concentrations during the kinetics between the colored species and **Ct**.

Another difference in both systems is the pH-dependent mole fraction distribution of the chemical species appearing at the equilibrium and the respective kinetics. When the plants need to increase or decrease the color to protect from the environment, anthocyanins have a reservoir of the colorless hemiketal (in fast equilibrium with *cis*-chalcone) that quickly increased or decreased the amount of color through the hydration/dehydration reaction (Scheme 7). In contrast, 3-deoxyanthocyanins have equilibrium between the colored species flavylium cation/quinoidal base and *trans*-chalcone (Scheme 8). The appearance/disappearance of color takes place through the interconversion of **AH^+^**/**A** and **Ct** and follows the kinetic curve of Figure 6a, whereas in anthocyanins it is the much faster hydration reaction that controls this process. 

### 2.8. The Colour System of Auronidins

**Scheme 9:**
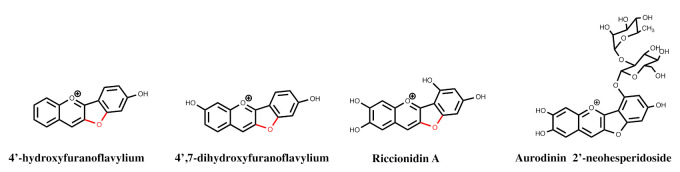
4′-hydroxyfuranoflavylium [11], 4′,7-dihydroxyflavylium [12], and riccionidin A, the aglycone of auronidin-2′-neohesperidoside [13], follow the same multistate of species of anthocyanins and 3-deoxyanthocyanins.

Auronidin-2′-neohesperidoside was extracted from the liverwort *Marchantia polymorpha* by Davies and Andersen [10], who named these molecules auronidins. In recent years, we studied the thermodynamics and kinetics of the so-called furanoflavylium compounds, a term used by Seshadri to designate the compounds shown in Scheme 9 [41].

**Scheme 10:**
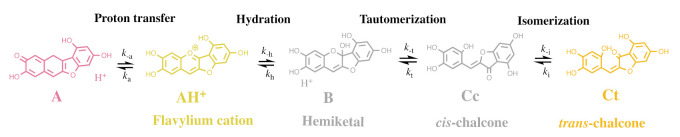
Riccionidin A follows the same multistate of anthocyanins and 3-deoxyanthocyanins [13].

We verified that as 4′-hydroxyfuranoflavylium [11], 4′,7-dihydroxyfuranoflavylium [12], and riccionidin A, the aglycone of auronidin-2′-neohesperidoside [13], follow the same multistate of species of anthocyanins and 3-deoxyanthocyanins (Scheme 10) [13]. Riccionidin A was identified in several liverworts, for example, in the Antarctic *Cephaloziella varians*, in response to an abrupt increase in UV-B radiation [42], in in vitro cultures of *Ricciocarpos natans* [43], and in adventitious root cultures of the Anacardiaceae *Rhus javanica* [44].

**Figure 7:**
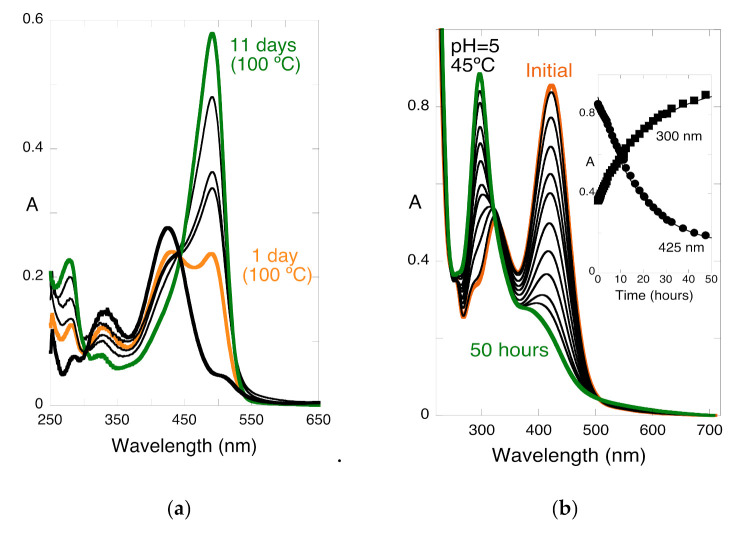
(**a**) Conversion of the *trans*-chalcone of riccionidin A in acetic acid saturated with HCl gas at 100 °C. (**b**) Spectral variations of the *trans-*chalcone at pH = 5.0 in methanol:water (1:1) at 45 °C, [Ct]= 5.0 × 10^−5^ M. The same behavior is observed in the pH range 1 < pH < 6. Adapted from [13].

The most interesting feature of riccionidin A is its extremely slow rate to convert *trans*-chalcone into flavylium cations. In acetic acid saturated with HCl gas at 100 °C, the conversion requires more than 11 days, Figure 7a [13]. At pH = 5.0 in methanol:water (1:1) at 45 °C, the reaction is again very slow, but the acidity is not enough to yield flavylium cations, Figure 7b.

**Scheme 11:**
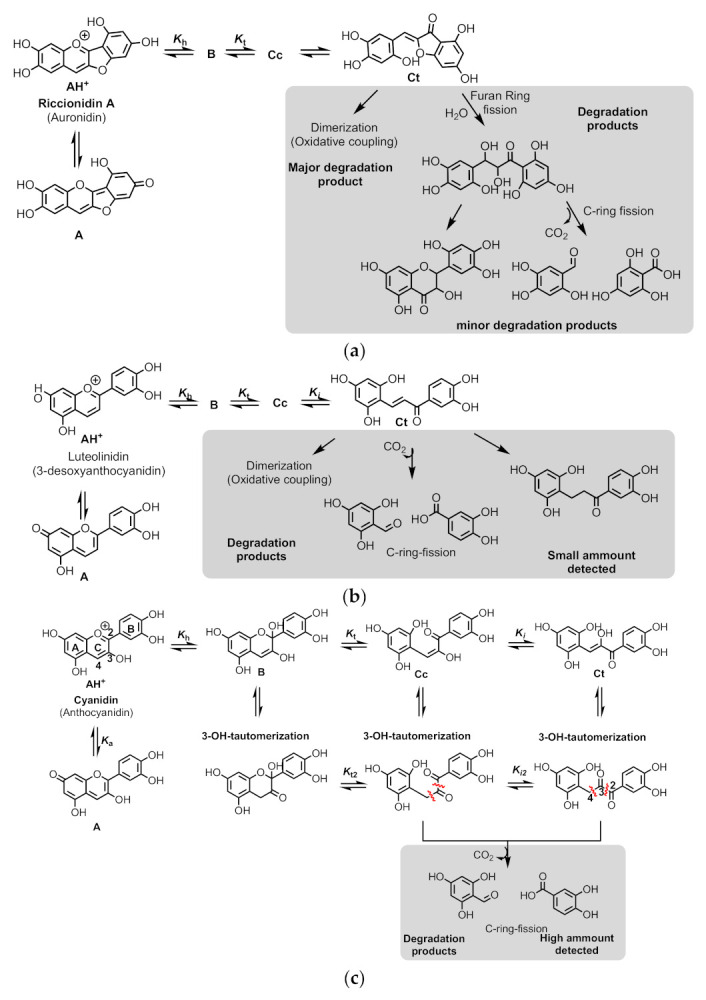
Thermal degradation pathways of (**a**) anthocyanidins, (**b**) 3-deoxyanthocyanidins, and (**c**) auronidins.

It was possible to identify the species **AH^+^**, **A**, **B**, **Cc**, and **Ct** by HPLC/MS [13] before decomposition (Scheme 11a) confirming that the multistate of species is the same as that of anthocyanins (Scheme 11c) and 3-deoxyanthocyanins (Scheme 11b). Moreover, the degradation products of these three families appeared to be similar (Scheme 11).

The degradation process of anthocyanidins starts with the keto-enol equilibrium involving C3-OH in hemiketal and chalcones species. The formation of the α-diketone species from the chalcones allows the easily oxidative C-ring fission of the molecule by the cleavage of the C2-C3 or C3-C4 bonds yielding two different degradation products (Scheme 11c) [39,45]. Glycosylation of C3-OH (anthocyanins) prevents this keto-enolic equilibrium and enhances the stability of the molecule. For anthocyanins, the degradation process starts with the breaking of the glycosyl bond releasing the OH group at C3 [45]. The system behaves from this point as an anthocyanidin. Moreover, the absence of an OH group at position 3 (3-desoxyanthocyanidins) also enhances the stability of the molecule, although some degradation product could be detected as a minor component (Scheme 11b) [46]. In this sense, the formed *trans*-chalcone is highly stable and its degradation by oxidative dimerization or by C-ring fission is much less extended than in anthocyanidins.

Auronidins also show a similar degradation behavior (Scheme 11a) [13]. In this case, the *trans*-chalcone is mostly degraded by oxidative coupling, and the dimerized species is the major degradation product detected by HPLC/MS. Moreover, the furan ring also undergoes a ring opening reaction that leads to degradation via C-ring fission as minor products.

The data previously reported by some of us [13] prove that riccionidin A follows the same multistate of species and chemical reactions of anthocyanins and 3-deoxyanthocyanins (Scheme 10).

**Scheme 12:**
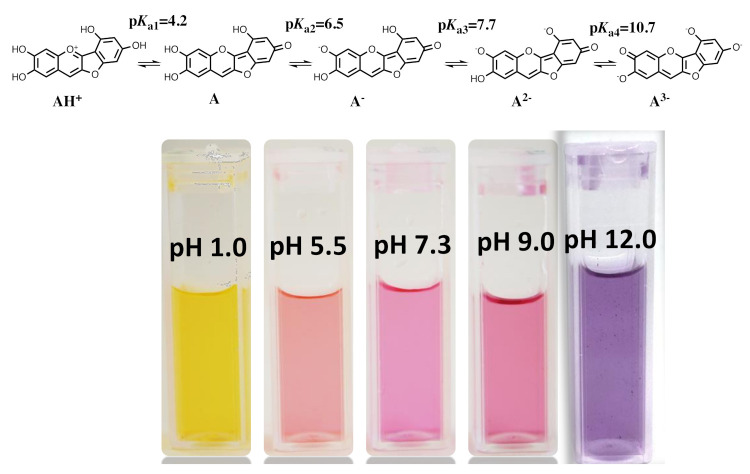
Proposed deprotonation sequence for flavylium cation of riccionidin A and the color pallet before decomposition.

The colors available in riccionidin A are shown in Scheme 12. The photos were taken immediately after a direct pH jump. The blue color was achieved only in extremely high pH values that are not found in plants.

### 2.9. Comparison between the Auronidins and the Other Systems

As described above in riccionidin A, and its glycoside form reported by Davies and Andersen [10], the flavylium cation is yellowish and the quinoidal base is red. The stability of riccionidin A is limited to pH < 4 and the rate constants between the species are extremely slow. At higher pH values, riccionidin A starts its degradation following a similar pathway as observed for anthocyanidins and 3-desoxyanthocyanidin. In this sense, these three families of compounds yield the same products in different extension depending on the relative stability of the chalcone species. Each of these three families were stable in acidic conditions but, when the pH increased, the mole fraction of the chalcone increased, and the degradation products started to appear in most cases by oxidative coupling or by C-ring fission degradation. As a suggestion for future work, it would be interesting to verify if auronidins are able to form associations by copigmentation or yield metal complexes, because there is a catechol in ring A that could link to metals. The furano ring prevents the attachment of the sugar in position 3 and the glycosylation occurs in position 2′. Synthetic flavylium compounds bearing a hydroxyl in position 2′ are able to form flavanones from the mono-anionic chalcones [47]. Another perspective for future studies would be to compare the Davies and Andersen auronidin-2′-neohesperidoside [10] with the results obtained for riccionidin A, and to identify the role of the sugar.

## 3. Experimental

### Thermodynamic and Kinetic Studies

The direct pH jumps were carried out by mixing a stock solution of kuromanin in HCl 0.1 M (3 × 10^−5^ M) with a solution containing NaOH 0.1 M and Theorell and Stenhagen universal buffer at the desired final pH using the stopped flow (SX20, Applied Photophysics; Surrey, UK) spectrometer equipped with a PDA.1/UV photodiode array detector. The direct pH jumps were also carried out by adding a small aliquot of a concentrated stock solution of the anthocyanin (pH = 1) into a 3 mL cuvette with water, with enough NaOH to neutralize the stock solution’s acid, and universal buffer at the desired final pH. Spectroscopic measurements were performed using Milli-Q water at a constant temperature of 20 ± 1 °C using Varian-Cary 100 Bio or Varian-Cary 5000 spectrophotometers. Reverse pH jumps were carried out by stopped flow (pseudo-equilibrated solutions) and common spectrophotometer (equilibrated solutions), adding enough HCl to reach pH = 1 in equilibrated solution of the anthocyanidin/anthocyanin at different pH values. The final pH of the solutions was measured in a Radiometer Copenhagen PHM240 pH/ion meter (Brønshøj, Denmark).

## 4. Conclusions

The physical chemistry of riccionidin A and 3-deoxyanthocyanins unequivocally shows that the former was significantly less versatile than the latter. Although anthocyanins are limited a priori to express color by themselves, they have a complete color pallet available and can give expression to these colors by means of intra- and intermolecular copigmentation, coordination with metals, and the combination of these effects, as in the case of metalloanthocyanins. It is clear that the color systems have evolved from auronidins to 3-deoxyanthocyanins to anthocyanins, by widening the color range they can cover. We hope the results reported in this work can contribute to the current discussion regarding the phylogenetic hypotheses about the divergence between mosses and liverworts, and between this group and vascular plants.

## Figures and Tables

**Table 1:** Equilibrium constants of kuromanin.

**p*K***′**_a_**	**p*K^^^*_a_**	**p*K*_a_**	**K_h_**	**K_t_**	**K_i_**
2.7	2.8	3.8	1.2 × 10^−3^ M^−1^	0.36	0.50
**p*K*_A/A_−**	**p*K*_B/B_−**	**p*K*_Cc/Cc−_**	**pK_Ct/Ct−_**		
6.15	7.8	6.8	6.5		
